# Supplementary material for: Liver X Receptor Activation Attenuates Oxysterol-Induced Inflammatory Responses in Fetoplacental Endothelial Cells
Source: Cells. 2023 Apr 19;12(8):1186. doi: 10.3390/cells12081186 (PMC10137015; doi:10.3390/cells12081186)
Supplement: Supplementary file 1 [file cells-12-01186-s001.zip › cells-2297930-SI.pdf]

S.1. 7-KetoC and 7β-OHC exert cytotoxic effects in fpEC at higher concentrations

Several oxysterols are known to induce cytotoxicity in a variety of cell types. Therefore, we aimed to investigate the dose dependent cytotoxic effects of 7-KetoC and 7β-OHC in fpEC by measuring the levels of LDH release after 24 hours of stimulation with different concentration of oxysterols (0, 1, 5, 10, 20, 50 μM). Both 7-ketoC and 7β-OHC significantly induced apoptosis in fpEC at 50 μM concentrations after 24 hours of treatment (Figure S1); 20 μM also appeared to be slightly but significantly toxic to the cells. The concentration we used for our studies (10 μM) was hence identified to be nontoxic to primary fetoplacental endothelial cells.

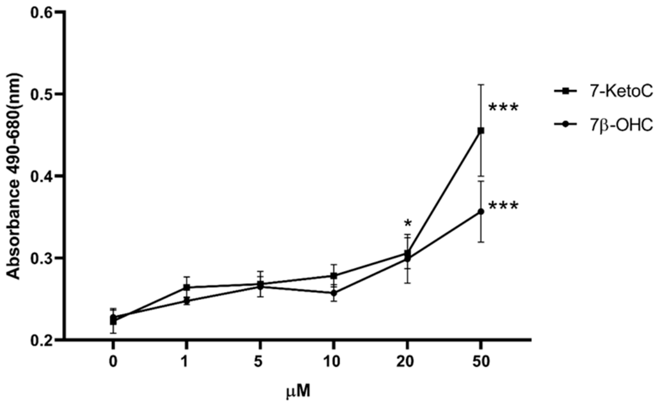

Figure S1: 7-KetoC and 7β-OHC exert cytotoxic effects in fpEC at higher concentrations. fpEC (n=5) in three technical replicates were grown on 96-well plates and treated with different concentrations of oxysterols for 24 hours. LDH was assessed in supernatants, and absorbance was measured at the wavelengths 490nm and 680nm, with the later used as the reference wavelength. Significant differences between the groups were calculated using two-way ANOVA, followed by Tukey's post hoc test. *p<0.05, **p<0.01, ***p<0.001.

S.2. Synthetic agonist T0901317 of liver X receptor attenuates oxysterol-induced inflammatory response in fpEC

Pre-incubation with 2 μM of T0901317 (TO) for 16 hours significantly reduced 7-ketoC and 7β-OHC induced IL-6, IL-8 and ICAM-1 mRNA expression. Total cellular ICAM-1 protein was also significantly downregulated in cells incubated with TO before the addition of oxysterols.

A **B**

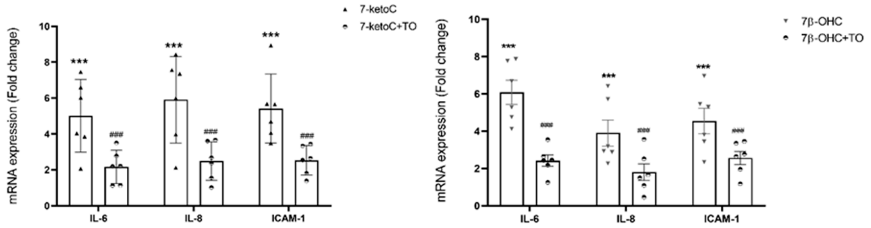

C

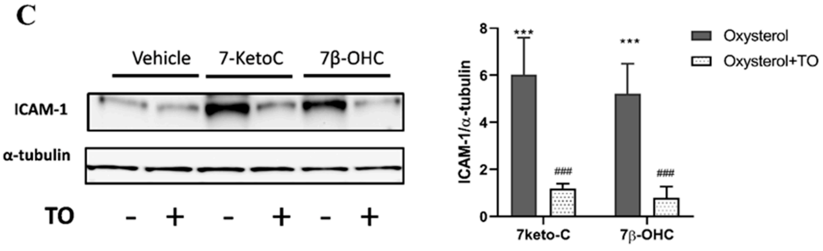

Figure S2. Synthetic agonist TO of liver X receptor attenuates oxysterol-induced inflammatory responses in fpEC**.** Cells (n=6) pre-incubated with TO displayed diminished 7-ketoC **(A)** and 7β-OHC **(B)** induced IL-6, IL-8 and ICAM-1 mRNA induction. The target gene fold expression in vehicle is normalized to one (not shown in the graph), and gene expression in all treatment groups is relative to the vehicle. **(C)** Representative western blot and quantitative analysis revealed reduction of ICAM-1 total cellular expression in cells pre-incubated with TO before the stimulation with oxysterols (n=4). Statistically significant differences between vehicle and oxysterol treatment (indicated with *) and between oxysterol and oxysterol + TO treatment (indicated with #) were calculated using two-way ANOVA, followed by Tukey's post hoc test. ###/***p<0.001.

S.3. LXR activation in fpEC is effective against LPS-induced inflammatory responses

LPS induced IL-6, IL-8 and ICAM-1 mRNA and ICAM-1 total protein expression was significantly reduced in LXR-activated cells.

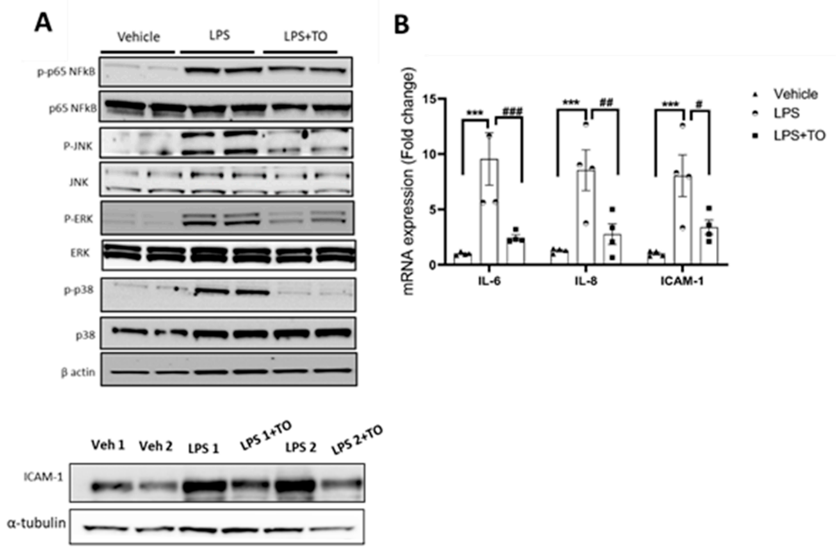

Figure S3**: (A)** LPS-induced MAPK and NFκB protein activation is reduced in LXR-activated cells. **(B)** LPS-induced IL-6, IL-8 and ICAM-1 mRNA induction is attenuated in LXR-activated cells. Statistically significant differences between vehicle and LPS treatment (indicated with *) and between LPS and LPS + TO treatment (indicated with #) were calculated using two-way ANOVA, followed by Tukey's post hoc test. #/*p<0.05, ##/ **p<0.01, and ###/***p<0.001.

S.4. Probucol antagonizes the anti-inflammatory effects of T0901317

Cells were pre-treated with 10 μM of probucol along with TO for 16 hours followed by 24 hours of 7-ketoC or 7β-OHC for cytokine and ICAM-1 mRNA and ICAM-1 protein quantification. As expected, probucol treatment induced re-induction of cytokine and ICAM-1 mRNA transcription and ICAM-1 translation to proteins.

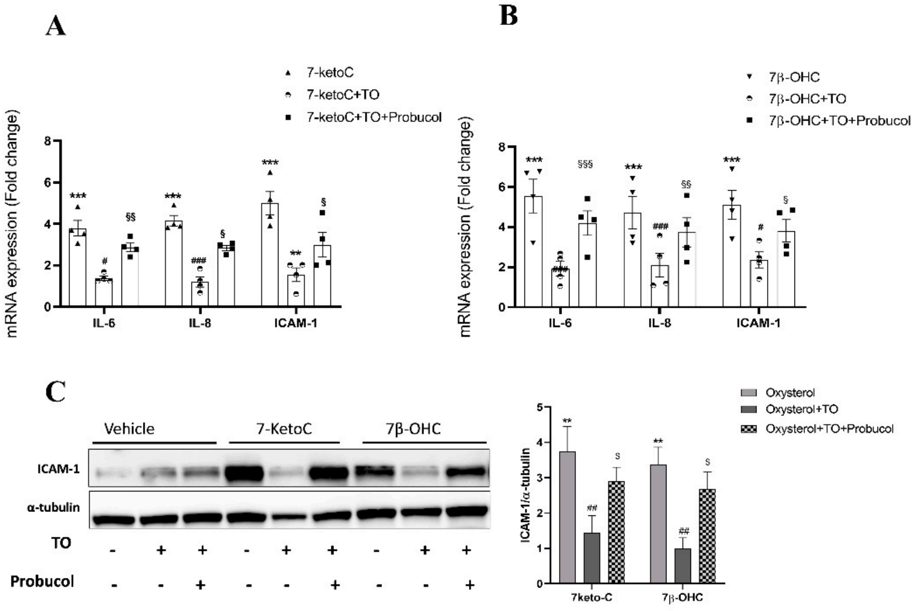

Figure S4: Probucol antagonizes the anti-inflammatory effects of TO in oxysterol-treated cells. Addition of probucol in fpEC induced IL-6, IL-8 and ICAM-1 mRNA despite the presence of TO in **(A)** 7-ketoC and **(B)** 7β-OHC treated cells. Similarly, ICAM-1 total cellular expression was also upregulated upon probucol treatment, as created in cells treated with oxysterol alone. Vehicle control is normalized to one (not shown in the graph), and gene expression in all treatment groups is relative to the vehicle. Statistically significant differences between vehicle and oxysterol treatment (indicated with *), between oxysterol and oxysterol + TO treatment (indicated with #), and between oxysterol + TO and oxysterol +TO + probucol (indicated with §), were calculated using two-way ANOVA, followed by Tukey's post hoc test. §/#/*p<0.05, §§/##/**p<0.01, and §§§/###/***p<0.001.

S.5. TLR-4 inhibition ameliorates oxysterol-induced inflammatory responses in fpEC

TAK-242 attenuated oxysterol-induced inflammatory responses in fpEC. Both elevated cytokines and ICAM-1 mRNA and ICAM-1 total protein levels were brought down by TAK-242. Tak-242 completely abolished LPS-induced inflammatory responses in fpEC, confirming the potential of Tak-242 to completely block TLR-4 signaling.

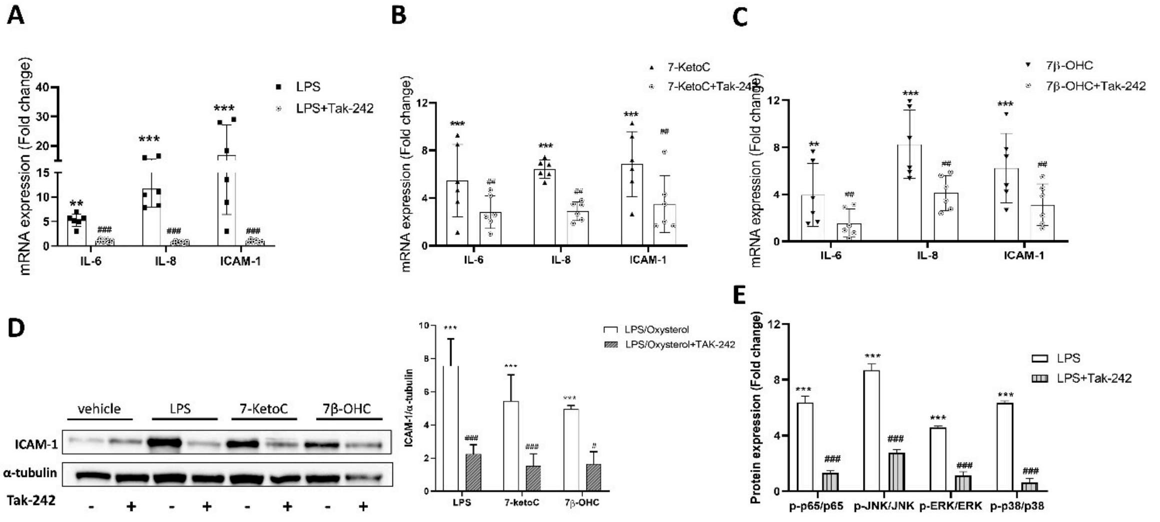

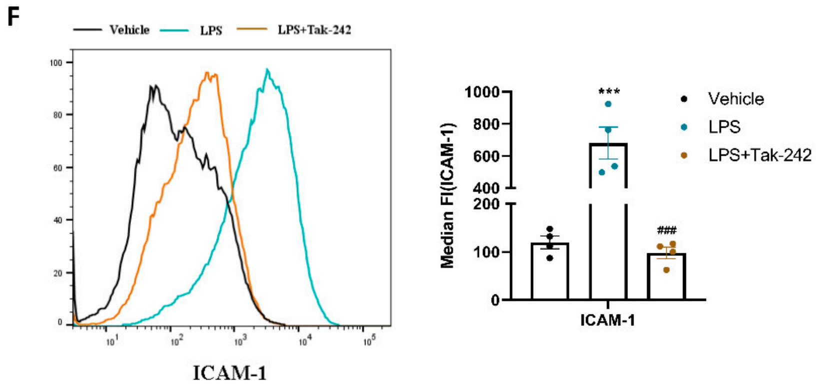

Figure S5: TLR-4 inhibition ameliorates oxysterol-induced inflammatory responses in fpEC. **(A)** Tak-242 treatment in cells nullified the LPS-induced cytokine and ICAM-1 mRNA in normal fpEC. Similarly, TLR-4 inhibition downregulated cytokine and ICAM-1 mRNA in 7-KetoC **(B)** and 7β-OHC **(C)** stimulated cells. Vehicle control is normalized to one (not shown in the graph), and all treatment groups are relative to the vehicle. LPS- or oxysterol-induced ICAM-1 total protein expression **(D)** and LPS-induced MAPK and NFκB activation **(E)** were also significantly reduced upon TLR-4 inhibition with Tak-242. **(F)** Tak-242 completely abolished the LPS-induced ICAM-1 cell surface expression, as indicated by FACS histogram and median fluorescence intensity plot. Statistically significant differences between vehicle and oxysterol treatment (indicated with *) and between oxysterol and oxysterol + Tak-242 treatment (indicated with #) were calculated using two-way ANOVA, followed by Tukey's multiple comparison test. #/*p<0.05, ##/ **p<0.01, and ###/***p<0.001.

Table S1: Subject characteristics

	Parameters	Control (n=12)
Maternal	Age (y)	32±6
	Height (cm)	165±4
	Pre-pregnancy Weight (kg)	63±7
	Pre-pregnancy BMI (kg/m^2)	23±2
	Weight at birth (kg)	75±8
	BMI at birth (kg/m^2)	28±2
Fetal	Gestational age (wk)	41±2
	Placental weight (g)	669±70
	Birth weight (g)	3114±482
	Length	50±1.5
	Ponderal index (g/cm3)	2.49±0.26
